# “They knew how to take care of people”: a qualitative study on older adults with chronic low back pain perspectives of an exercise plus education program

**DOI:** 10.1186/s12998-025-00586-z

**Published:** 2025-06-04

**Authors:** Fabianna Resende de Jesus-Moraleida, Ana Carla Lima Nunes, Crislaine Silva Costa, Lara Figueiredo Vasconcelos, Nathalia Costa

**Affiliations:** 1 Master Program in Physiotherapy and Functioning, Federal University of Ceará, Fortaleza, Ceará Brazil; 2 Department of Physiotherapy, Federal University of Ceará, Fortaleza, Ceará Brazil; 3 The University of Queensland’s Clinical Trials Capability (ULTRA) Team, Centre for Clinical Research, The University of Queensland, Brisbane, QLD Australia

**Keywords:** Aging, Low back pain, Primary health care, Exercise therapy, Pain education, Qualitative research

## Abstract

**Background:**

Exercise therapy and education are first-line care for those with chronic low back pain (CLBP), but information on its applicability in older adults is limited, and adherence to non-pharmacological treatments in this population is challenging. This study explored perspectives of older adults with CLBP on a group-based exercise plus education program supported by text messages—PAT-Back.

**Methods:**

This descriptive qualitative study was embedded in a feasibility randomized controlled trial (RCT) with older adults aged ≥ 60 with CLBP who participated PAT-Back. PAT-Back consisted of pain education, group and home-based exercises, with the latter being supported by text messages. After the feasibility RCT finished, we conducted 14 (13 women, 1 man) semi-structured interviews with participants. Interview questions were designed to elicit participants’ reflections on the program, including its impact on symptoms and factors that facilitated and hindered participation. Transcripts were thematically analyzed.

**Results:**

We identified four themes related to participants’ perspectives on PAT-Back: 1) *It takes more than intrinsic motivation*: Participation in the PAT-Back program was linked to family and peer social support, clinician’s supervision, caring clinicians and inaccessibility to treatments; 2) *PAT-Back exceeded expectations*: Before the intervention older adults did not expect or believed exercise would reduce pain, but this changed as they experienced improvements while engaging in the program; 3) *Coexisting comorbidities, challenges with mobility and transport got in the way*: at times, having pain in other body sites and not having access to transport and/or safe environments hindered exercise participation; 4) *Technology can be both helpful and challenging:* motivational text messages were perceived as beneficial by some, but others highlighted challenges related to access and technology literacy.

**Conclusions:**

Older adults’ experiences in exercise and education programs for CLBP may be enhanced by social support (from clinicians, peers, family members), access to healthcare and embodied experiences of improvement. Such experiences can also be hindered by comorbidities and broader factors, such as safety to exercise outdoors. Exercise and education interventions for this population should be tailored at the conception phase to meet their needs, especially regarding social support and environmental infrastructure to promote participation and improve symptoms.

## Introduction

Chronic low back pain (CLBP) is the leading cause of disability worldwide, with older adults bearing a significant burden [1]. Exercise therapy is effective in reducing pain and disability compared to no treatment or usual care [2, 3] and therefore, it is considered first-line care for those with CLBP [4]. In 2024, the World Health Organization (WHO) released an international guideline to provide evidence-based recommendations on non-surgical interventions for CLBP in adults (including older adults) to promote interventions that can be delivered in primary care and community settings [4]. The WHO guideline for CLBP recommends both exercise and education for CLBP, but also highlights that the overall certainty for these treatments in older adults is very low, emphasizing the need to build robust evidence to increase the level of certainty [4]. Literature evaluating the benefits and harms of interventions for older adults with CLBP is still limited [4–8]; therefore the Guideline Development Group commissioned a qualitative synthesis of primary qualitative studies on older adults’ perspectives of treatments for CLBP to gain additional insights into how different interventions were considered acceptable by older adults. Notably, only 22 studies were included in the qualitative synthesis. While some of them provided evidence that some interventions are acceptable (e.g., education, topical Cayenne pepper), none of them provided insights into older adults’ acceptability of exercise therapy for CLBP, highlighting the need to build evidence in this area [4].

It is not surprising that the WHO’s guideline for CLBP highlights the need to develop further trial-based and qualitative evidence for interventions targeting older adults. Although researchers have been advocating for the development and testing of interventions for older adults for over a decade [9], research targeting interventions for this population is still lacking. Most RCTs testing interventions for CLBP exclude individuals over 60 years [10]. Notably, this group presents distinct features compared to the general population, such as a higher prevalence of comorbidities alongside pain, higher risk of falls [11] and greater side effects due to interactions of medications and polypharmacy [12]. These and other factors experienced by older adults could pose major challenges to adhering to conservative care and decreasing disability associated with CLBP. The dearth of evidence supporting interventions for older adults can prevent clinicians and policymakers from implementing high-quality care to improve outcomes for this population, both at individual and population levels. While this is a major challenge globally, low- and middle-income countries likely endure a greater burden than high-income countries due to limited resources, and both the faster and larger population ageing [13]. When combined, these contextual factors may widen the gap between evidence-based care and the care provided to older adults, as well as the gap between the burden of CLBP across low-, middle- and high-income countries [14].

In an attempt to contribute to the literature on interventions for older adults with LBP, we assessed the feasibility of conducting a randomized controlled trial (RCT) comparing the effectiveness of an eight-week exercise plus education program (PAT-Back) to a single best practice advice consultation, and considering pain and disability in older adults with CLPB [15, 16]. Our results revealed that the RCT was feasible—retention rates were 100% in the intervention group, and 95% in the control group, and the safety of performing exercises was partially acceptable (70%) [16]. Almost all (95% of participants) reported that the text messages were useful for encouraging participation in home-based exercises [16].While adherence rates were 60%, only six participants reported minor transient adverse events (e.g. transient flare of symptoms after exercises). Both the study protocol and the findings were published elsewhere [16] and the process of implementing this feasibility RCT highlighted the need to explore older adults with CLBP perspectives on participating in an exercise plus education intervention.

Qualitative research embedded in RCTs can deepen our understanding of our results for this population [17], helping to optimize interventions and shed light on the experience of LBP in older adults, alongside barriers and facilitators that must be considered when delivering these interventions [18]. We interviewed study participants after the completion of the feasibility RCT to explore their perspectives on PAT-Back and understand the impact of the education plus exercise intervention on their symptoms, as well as factors that facilitated and hindered participation (e.g., potential challenges associated with home-based exercises and use of text messages). We sought to build evidence related to older adults’ acceptability of interventions for CLBP by addressing the following research question: “What are the perspectives of older adults with CLBP on an exercise plus education program (i.e., PAT-Back)?”

## Methods

### Study design

We employed a descriptive qualitative study and gathered data through interviews. We chose a descriptive approach because we wanted to prioritize participants’ own language and meanings when summarizing patterns across the dataset [19]. Relatedly, we conducted semi-structured interviews with PAT-Back participants because this method of data collection is well-suited for exploring individuals’ perspectives and experiences with a range of phenomena, including healthcare interventions [20]. Of note, this study was conducted from February to May 2022 upon completion of a feasibility RCT with older adults aged ≥ 60 with CLBP who participated in an exercise plus education intervention supported by text messages (PAT-Back) [15, 16] (see Box 1 for details about the intervention).

**Box 1:** Characteristics of participants

Individual consultation
• Best practice advice based on an educational booklet (e.g., understanding low back pain, importance of being physically active, gradual exposure to activities, planning, and pain self-management strategies) • Identification of baseline exercise level, functional limitations and individual targets to be considered in both group exercise sessions and home-based exercises
Intervention
• 90-min group sessions for 8 weeks, plus a home-based exercise program, both based on the findings of the initial individual consultations • Patient education including self-management of pain and comorbidities, pain-related beliefs and coping, sleep quality, stress reduction strategies including distraction, recreational physical activity, and activity pacing. Mobility and strength exercises targeting the lumbo-pelvic region and lower limbs (e.g., pelvic tilt, walking, sit-to-standing, hip abduction, truck extension, step-up and down exercises), relaxation, and walking training • WhatsApp messages (3 times/week) reminding and encouraging the patient to engage in the exercise program (e.g. reminder of benefits of becoming active, incentive to exercise, incentive to set goals, encouraging self-management implementation) • Individualized exercise progression based on cognitive behavioral therapy principles (e.g. reflections on dysfunctional beliefs and behaviors, emphasis on collaboration and active participation, shared and meaningful therapeutic goals, structured and time-limited intervention)

The PAT-Back feasibility RCT study derives from a post-positivist paradigm [21], which assumes we can approximate the objective truth regarding the effectiveness of treatments. Recognizing that different paradigms offer valuable insights and that no single paradigm can capture all facets of a phenomenon [22], this paper aims to add to the knowledge regarding older adults’ experiences with, and acceptability of, an exercise plus education intervention for CLBP by drawing from interpretivism, which posits that different individuals experience and understand reality in various ways [23]. The study was approved by the Ethics and Research Committee of the Federal University of Ceará (ethics 4,565,335).

### Researchers’ reflexivity

Reflexivity is the process and practice of a researcher reflecting on how their disciplinary and personal experiences shape the design of their study, delimiting the knowledge they produce [19]. This concept denies the idea of knowledge as objective and independent from the researcher [19]. To this end, the design of this study and our analysis are influenced by our interests in older adults with CLBP and social justice, given that this population tends to be under-researched and underserved. We also draw from our experiences conducting research and working clinically in low-resource settings in Brazil, meaning that our analysis was sensitive to social aspects embedded in participants’ responses (e.g., scenarios where low socioeconomic status and low digital literacy were evident). None of us has CLBP, although all of us have had episodes of LBP. Two of us were also involved in the feasibility RCT (FRJM, ACLN), meaning that our readings of participants’ experiences often overlapped with our own experiences conducting that study.

### Participant recruitment

We invited all participants from the intervention arm PAT-Back to participate in the interviews. Participants of the feasibility RCT were adults 60 years and older with non-specific CLBP who had mobile phones, scored three points or higher in the Start Back Screening Tool (SBST) and lived in Fortaleza, a city in Northeast Brazil with a Human Development Index (HDI) of 0.75. The specific area where the study was conducted had an HDI is 0.490, meaning the location of this investigation was an underserved area of Fortaleza. Exclusion criteria included: i) severe cognitive impairment; ii) having a diagnosis of specific low back pain (e.g., fracture or stenosis); iii) neurological or systemic diseases in the acute or decompensated diseases; iv) rheumatic diseases (e.g., fibromyalgia); v) history of thoraco-abdominal surgery in the previous 12 months, vi) history of spine surgery, vii) having undergone physiotherapeutic treatment in the 12 months before data collection, viii) severe visual deficits and ix) presence of contraindications or limitations that prevented walking for at least 10 continuous minutes with or without walking aid devices. Participants of the feasibility RCT were recruited through health centers, places with a large circulation of people (e.g., primary care centers/university halls and clinics), social media (e.g., Instagram) and posters on buses. Data collection for the primary study took place from September 2020 to January 2022, in Fortaleza.

Upon the completion of the feasibility RCT, we invited all participants from the PAT-Back intervention group (N = 17) to participate in the interviews. We contacted participants directly via phone calls and text messages. From the 17 participants, two refused to participate and one did not attend the interview even though the interview was organized at a time convenient to them. The remaining 14 participants agreed to participate and were interviewed. While we aimed to include a diverse sample in the present study, the diversity of our participants was limited by the diversity of the participants of the feasibility RCT. Still, we invited all participants to participate in the interview, with the view of recruiting individuals with diverse schooling and income levels (self-reported mean salary in Brazilian reais, where 1 real equals approximately 0.18 US dollars), ages, pain intensity (measured on a 0–10 numerical rating scale), self-reported number of comorbidities, and intervention adherence [24]. As only one man participated in the feasibility RCT (and agreed to participate in the interview), the gender diversity of our sample was limited. All participants provided verbal consent prior to participating in the interviews.

### Data gathering

Interview questions were created by FM, ACLN, and NC and pilot-tested twice by the LF and JS (see Appendix 1). NC is a Senior Research Fellow in Qualitative Methods with growing experience in applying such methods to RCTs. She trained the research team in the basic principles of qualitative methods, interviewing and thematic analysis. NC listened to the pilot interviews, provided feedback, listened to the interviewers’ reflections, discussed these with the team and they refined the interview guide accordingly. We used primarily open-ended questions to incite discussions about participants’ experiences with PAT-Back, with prompts when necessary. Interviews were scheduled with those who provided consent to participate at a date, time, and location convenient to them. Interviews were conducted using the mode preferred by each participant, i.e., in-person (at the Department of Physiotherapy) or via teleconference software (Google Meet). The interviewers were both novice researchers interested in healthy aging and received training in conducting interviews–JP, a physiotherapist, and LM, a Physiotherapy undergraduate student who held a scholarship during the study. They were also trained in assisting participants to join the teleconference software if they reported being unfamiliar with the it. JP and LM were not involved in delivering the interventions and had no previous relationship with participants before interviewing them, although participants knew they were involved in the feasibility RCT research team and were interested in exploring their perceptions about the intervention. Both interviewers wrote field notes upon completing the interviews, where they highlighted their overall impressions of how the interview went (e.g., reflections on what they did well, opportunities for improvement) and what stood out to them (i.e., aspects that were insightful in addressing the research question). The interviews were recorded by audio recording and transcribed verbatim by LM and CS, who deidentified and checked all transcripts to ensure confidentiality and accuracy.

### Data analysis

We analyzed the interview data using a codebook approach to thematic analysis [19] because: i) we wanted to describe patterns descriptions of their experiences with the intervention; ii) qualitative research was new to most team members (all but NC). Within this context, the flexibility and accessibility of the codebook approach to thematic analysis can make an introduction to qualitative research relatively simple [19]. To this end, each team member read three interviews in their own time, taking notes of things that stood out to them. Their initial impressions were then discussed in one 2-h research team meeting, whereby the team members’ impressions were used as a foundation to develop initial codes. After that meeting, FM coded two interviews using the comment function of Microsoft Word and drawing from the initial codes discussed amongst the team, as well as codes inductively developed throughout the coding process. The two coded interviews were then reviewed by NC, with the review to ensure that the codes were informative and descriptive rather than simply categorical (e.g., ‘cost as a barrier’ rather than simply ‘barrier’), as well as ensure that both semantic (i.e., explicit, surface meaning) and latent (i.e., implicit, underlying meaning) were included [19]. All codes developed in this initial coding process were then transferred to an Excel sheet and used as a codebook to guide the coding of remaining interviews, which was conducted by CS and LM, who coded 6 other interviews each. Both CS and LM added to the initial list of codes as they progressed with the coding of other interviews. FM, ACLN, and NC then met to discuss the codes and their reflections on them. In that meeting, they also combined the codes into broad preliminary themes under the guidance of NC. The theme candidates were then presented by the other co-authors for their input and refined throughout the writing process. Results are reported according to guidance for thematic analysis [25].

## Results

### Characteristics of participants

We interviewed 14 participants (13 women) with a range of education levels and baseline pain intensity scores, with ages ranging from 61 to 84 years (see Table 1 for individual descriptions of participants).

**Table 1:** Characteristics of participants

Participants	1	2	3	4	5	6	7	8	9	10	11	12	13	14
Codename	Rita	Helena	Célia	Maria José	João	Lúcia	Sônia	Ana	Tereza	Claudia	Francisca	Fátima	Joana	Antônia
Sex	Female	Female	Female	Female	Male	Female	Female	Female	Female	Female	Female	Female	Female	Female
Age (years)	61	61	65	66	75	70	62	74	61	64	84	64	63	76
Highest level of education	Secondary School	Secondary School	University degree	Primary	University degree	University degree	University degree	Secondary School	Primary School	Secondary School	Primary School	Secondary School	Secondary School	Primary School
Monthly income*	Less than 1 salary	More than 3 salaries	1–2 salaries	1 salary	1–2 salaries	1 salary	1–2 salaries	1 salary	Less than 1 salary	Less than 1 salary	More than 3 salaries	1–2 salaries	More than 3 salaries	1–2 salaries
Number of dependents	2	3	1	3	4	1	1	3	5	1	4	3	2	4
Self-reported baseline back pain intensity (Numerical Pain Rating Scale [NPRS] - 0-10)	9	7	6	8	3	8	10	8	10	6	4	5	9	3
Self-reported baseline leg pain intensity (NPRS)	9	5	9	9	0	7	8	6	10	3	0	0	9	4
Number of self-reported comorbidities	5	7	2	2	3	2	2	1	5	4	5	4	4	4

Monthly income based on 1 Brazilian income salary (2022): R$ 1.212; based on 0–10 numerical rating scale

The average length of the interviews was approximately 35 min. Our results are presented in four main themes that explore their experiences with PAT-Back, including their experiences with exercise and views regarding the text messages they received to prompt engagement and provide support regarding home-based exercises. We describe the themes below alongside data excerpts to illustrate each theme and use pseudonyms to anonymize participants. Table 2 summarizes the themes and provides additional illustrative quotes.

**Table 2:** Summary of themes and illustrative quotes

Theme *Theme description*	Illustrative quotes (additional to the ones highlighted in the text)
It takes more than intrinsic motivation *Participants’ motivation to sign up for PAT-Back, attend group-based sessions and do the home-based exercises was often linked to support from others (family members, peers, physiotherapists), their pain intensity and the inaccessibility of interventions for CLBP, which made them see PAT-Back as an opportunity to access care*	*“They [the physiotherapists] were dedicated, caring…I loved [it].”* Francisca *“I never had that, a physiotherapist, someone giving me advice and attention, explaining to me what I had to do. I never had that. I couldn’t afford it as it requires a payment. (…) Like they say—it was a blessing because it is difficult and expensive.”* Tereza *“I hope the project continues to treat these people who need it, because it is not easy—it is not easy to depend on the public system, it is not easy. You go on a waiting list for two, three years and you don’t leave the list. And a project like this can help a lot. How long did it last for? Eight weeks? But eight weeks where we worked hard, you understand? [Participant’s name] did not miss any session, I didn’t miss any session, and I got benefit from it. [Another participant’s name] also did not miss any of the sessions.”* Rita
PAT-Back exceeded expectations *Participants were initially skeptical about the role of exercise for managing CLBP, but embodied experiences of improvement through movement and educational information shifted their perceptions. For some, perceptions about the role of rest, medication and scans also shifted. As a consequence, they expected to participate in the program for a longer period of time or indefinitely*	*“I thought it was superfluous [exercising], I didn’t like it. Now that I’ve done it, I think it’s worth doing. I’ve improved, I enjoyed doing it. But I’m the kind of person who needs encouragement* Joana *“In relation to the exercises I did here, I’m doing much better, I’m able to work more. I work with handicrafts, I sit for a long time, so I couldn’t sit for long anymore because the pain was so bad in my back and here in my lumbar, here in my hip. So, after I took part in this experience of yours, with the exercises I improved a lot because I learnt how far I can take that pain, what’s normal, until I can take it and what’s not normal.”* Cláudia *“I didn’t think it was going to be so short, I thought we would participate in it for longer…like for the rest of our lives. But then I was informed that it was just a project, so I understood it was a project situation.”* Lúcia *“So we always had a chat, this chat, after a ‘psychological warm-up’, motivational…it wasn’t only psychological, it was motivational, do you understand? I learned how I was uneducated about my body, how I was treating myself badly. The way in which we were exposed to education was very rich in terms of helping me understand how someone was and how someone could be…and the path that needs to be taken.”* Célia *“I thought it was going to worsen, my pain, that it would become more intense, that I wouldn’t be able to do certain movements but with the treatment and the instructions from the physiotherapist everything turned out to be different from what I imaged.”* Rita *“I went to the program feeling hopeful and, like, it met my expectations, Thank God. (…) Because I couldn’t do anything, 10, 15 days lying in bed without moving with a really stiff back. I used to go the Emergency Care Unit, take medication and go home, within 30 min I was in bed again…”* Fátima *“I expected to improve, but not so fast. (…) When I started to do [the exercises] I got sore, but not sore on the lower back. I felt sore on my body, which is too heavy. My legs were sore, I was broken, Jesus Christ! On the other hand, I would move and then the pain…I didn’t have pain anymore, despite having just started. And from there onwards it just got better and better, until now.”* Tereza *“My only suggestion is that the program should continue. Too many people need it.”* Ana
Coexisting comorbidities, challenges with mobility and transport can get in the way *Participants discussed comorbidities (including pain in other body sites) as important barriers to engaging with PAT-Back and exercise more broadly. At times the weather, transport, lack of accessible and safe environments also impacted participation*	*“Also [have difficulty in] walking, because I have a high degree of arrhythmia and I get tired (…) I’m not going to say that I do it [exercise] regularly, because I don’t. I can’t even walk, because I get tired. If I walk two blocks I get tired, so I can’t do it.”* Antônia *“The time of the sessions was a bit inconvenient because I had to leave at 1 pm.”* Francisca *“The only difficulty was walking from there all the way to my place. It was a long distance, it was far.”* Tereza
Technology can be both helpful and challenging *While the text messages were helpful for some, others reported difficulties in reading and/or responding to them due to low digital literacy or because they did not have the habit of using their mobile phone for purposes other than making phone calls*	*“No, it’s not liking, it’s forgetting [to look at the phone]. Whereas, for example, I have a grandson who takes your mobile phone and does everything in the world very quickly. And I wasn’t born for mobile phones. When you call me, I answer. That’s it, or I’ll leave it here.”* João *“Sometimes you think, I’m not going to do it now, I’ll leave it for later. Oh, I’m not going to do it today, they’re not really watching, you know, I’m not going to do it. But when you’re about to say no, [you think] they’re already wanting to help me, and me not doing it, that’s not right. So, I think this is a very important thing: they helped me a lot, they encouraged me to do it, they were the ones who never forgot ‘do the exercise’. It’s an incentive that the person is giving us.”* Ana *“Sônia: The text messages influenced a lot [whether I did the exercises]!* *Interviewer: How…can you tell me more about it?* *Sônia: Well, when I was starting to forget about them, I would get a message saying ‘we have a session tomorrow, don’t forget to do your exercises’. It was a good reminder for me.”*

### Theme 1: it takes more than intrinsic motivation

A few participants discussed that their participation in the exercise plus education program was influenced by self-motivation and a sense of responsibility. Yet, these factors were not always brought up when participants discussed what made them sign up for PAT-Back and continue to engage with it. Overwhelmingly, most participants emphasized how family and peer social support, clinicians’ supervision and their caring nature, the intensity of their pain, and the constraints related to accessibility to healthcare were the main reasons why they engaged with PAT-Back. Collectively, these aspects seemed to impact motivation to join PAT-Back, attend in-person sessions and engage with the home-based exercises independently. The excerpt below illustrates the perceived relevance of family support for signing up for the program:

*“My daughter is an exercise physiologist, and she encouraged me a lot, right? I told her and she said ‘mom, go and don’t give up, don’t give up because you will learn to cope with pain there’.”* Fátima

Although the participant herself was unsure of how to cope with long-term pain, her daughter’s encouragement facilitated the first steps towards engaging with the program and then sustaining her participation over time. Contrastingly, the lack of social support was discussed as a barrier to continuing to be engaged with the exercise program, especially given their age and their perceived need to have emotional/physical support from peers/carers:

*“One more thing, if I had a companion to go with me, I could ask him and carry on, doing it, but at the moment I can’t, I’m not really one for disturbing anyone, you know? I’m an old lady.” Antônia*

Relatedly, the support from the group encouraged participants to engage in the exercise program even when in pain or when aspects of their personal lives were not going well. Within this context, participants emphasized how the group format of the program fostered peer support and promoted a sense of ability to perform exercises through camaraderie:

*“No, I don’t like [exercising at home on my own]. Because when we go to the group, doing it with the group, it’s more lively, right? We talk, we get less tired. Doing it at home makes me more tired. I’m already 70 [years old]. And doing it in the group is wonderful, I prefer the group. I don’t like doing it alone”. Lúcia*

Similarly, the support provided by the physiotherapists who delivered the intervention was also valued by participants and seemed to facilitate their engagement with both supervised and home-based exercises:

*“It was their encouragement (...) they wouldn’t let us stop the exercises. If I said, ‘I can’t’, they’d say ‘you can do it, I’ll tell you how to do, I’ll keep supporting you, don’t give up’. It was their incentive [that made me do it], as I can’t do it alone.” Helena*

Notably, a few participants appreciated the support, but felt they were self-responsible for their participation and progress. Yet, this perspective was not shared by most of them:

*“It’s like, I was at school, I had to learn that and with the physiotherapist, I was doing it, she was teaching me the right way [to do it], I had to take advantage of it and I managed to get the way she was teaching me so I could have the best result [...] then it’s something that you get there and you go and do, you see that you need it, you see that it’s the right thing, then you go and do it...you have that responsibility to do it and to this day I still do it.” Ana*

Participants also highlighted pain intensity and accessibility to healthcare as a noteworthy concern that prompted them to sign up for PAT-Back. They expressed that their motivation to join the program came from the opportunity to access treatment, which would otherwise be difficult due to the inaccessibility of appropriate care within the public health system. They shared that opportunities for treatment are scarce, and discovering the program through social media provided an opportunity to receive the support they needed:

*“[What made me join the study] was the need to improve my quality of life because until then, I didn’t know where I could get treatment. We look for Unified Health System [Brazil’s public health system] and there’s never treatment available, so when I saw it [the study advertisement] on social media, I decided to apply and thank God I was selected for my own good and I’m good [now].”* Rita

Similarly to Rita, Ana also expressed how the program offered care while providing encouragement, emotional support, and a sense of being valued–something she found meaningful given the challenges older adults face when accessing healthcare services. She shared how older adults are often marginalized when in need of support:

*“So it was that encouragement just to take you with them, to lift you up, to give you support. So I thought this was very important, because nowadays it’s very difficult for people to want to help people, especially when it comes to old people, right? It’s always the old, the children and elders who are left behind, and this group was a group that tried to help, they knew how to take care of people and that I’ll never forget that care, that affection.”* Ana

Taken together, these examples suggest that engagement in programs targeting low back pain is shaped by various contextual factors, including attitudinal (e.g., sense of responsibility), relational (e.g., support from others) and systemic factors (e.g., [in]accessibility to treatments). The excerpts also convey the perceived benefit of and preference for group-based activities. Many participants believed that exercises came with difficulties, but social interaction and collective support were crucial in the program. Overall, participants’ engagement with PAT-Back was facilitated by factors beyond intrinsic motivation.

### Theme 2: PAT-Back exceeded expectations

Overwhelmingly, participants discussed the program as an intervention that led to meaningful improvements and exceeded their expectations. For instance, many participants discussed that up until they engaged with the program, they did not think exercise could improve their pain, particularly because of their age. A few discussed that previous advice from health professionals prevented them from doing physical activities and made them rely on rest and medication for pain relief. Participants also discussed how previous experiences influenced their beliefs about their prognosis and how experiencing improvements during the program changed their views. For instance, Lúcia reflected on what she learned during the education sessions—that is, that current evidence suggests scans are not helpful for LBP:

*“And thank God I learnt to cope because I was even going to have an MRI scan of my spine, which the doctor had ordered, and I didn’t even have to pay for it, you know? I couldn’t get it through the public system, that’s expensive too, right? So I didn’t do it.”* Lúcia

Likewise, Fátima expressed skepticism about the exercise protocol as it seemed too simple. Given her extensive history with pain and previous experiences with different treatments, she found it difficult to believe that basic education plus exercise could help improve her condition:

*“Because, well, how something so simple is going to solve this problem, this chronic pain that I’ve been feeling for I don’t know how long? I’ve taken so much medicine, so many things and it didn’t go away and the pain will go away with this? We don’t really believe in it, do we? We lose faith when we had pain for a long time (...) As I said, it was a great surprise for me. To be honest I did not think it was going to work (…) I was very afraid of any movement, I would feel pain there (...) then I’d go to bed, lay down and lay down for several days, being stuck, without being able to move from side to side. And I lost that fear. (…) I thought those simple exercises would not solve my problem, but I was really wrong”* Fátima

The skepticism faded as participants experienced clinical improvements while engaging in the program. Indeed, engaging in education plus exercise seemed to shift their perception regarding the benefits of exercise for improving their condition. For example, Sônia highlighted the positive impact the education plus exercise program had on her CLBP:

*“Well, I thought it was impossible to do the exercises when I was in such pain. And I didn’t really trust it. I think completely differently [now]. Because I did the exercises, I took part in the group, it was really good. But yeah, I think differently, really differently. And when I started doing it, I saw that I really improved a lot. I didn’t really trust it would work, I thought it was something that might not work, but it worked for me.” Sônia*

Célia shared her experience in joining the program and perceiving its benefits. She reported giving up on self-medicating and started doing exercises for analgesia, emphasizing a growing sense of capability:

*“Ah, I’m getting old. Let it go, it’s over, right? It’s over! It’s not, we can still give so much, you know? And that was one of the things I took into my life. People like me can do something. People like me can walk. How well I can sit. People...[pause] I’m off medication, it’s unbelievable, you know?” Célia*

This was not unique to Célia. Other participants shared reflections on initial skepticism about the effectiveness of exercise (particularly related to concerns about exercise exacerbating their pain), and discussed how the program has impacted their lives, promoting both a shift in thinking and symptom improvement. Yet, although the program was seen as beneficial, some participants expressed that it would have been better if it had lasted longer. After the group ended, participants reflected on the positive impact it had on them, and many expressed a desire for ongoing or longer participation:

*“So, for me, it was a very rich, very good experience, you know? It made me aware of a lot of things, because of this program of yours, I was able to see how complacent I was. You know? You gave us a lot of exercise. So, it was a marvelous awareness and I brought it home to myself with your very good support, you know? You took me away from being complacent and we’ve seen a really good effect, but it’s a pity it wasn’t for long. It would have been great if it had been longer, right? But the experience is valid, you must continue, you know? And I’m sure it has only brought me benefits.” Célia*

One participant also shared that while her experiences with the PAT-Back were largely good, having psychological support as part of it would have been very valuable. She noted that some of her peers brought up personal challenges during PAT-Back sessions, which prompted her to think that psychological support would have been valuable:

*“The only thing I realized there is that many of us have problems at home, and in addition to this, we realized that many of the causes are also due to the fact that you have a very hectic, busy life, and there’s a lot of conflict at home. (...) Sometimes there’s a problem with a husband, with a child, all of which means you don’t sleep properly (...) Psychological support for these women during the group activities would also be ideal, right? (...) some people tell us about it, about their child using drugs, all that stuff (...) They get ill because they don’t sleep, they don’t eat, they worry a lot, they run the risk of having a stroke, a lot of things, right? So (...) psychological support would have been also ideal.” Rita*

Taken together, these excerpts highlight participants’ experiences with PAT-Back were largely positive. Yet, being able to participate in the program for longer periods of time and receive psychological support alongside education and exercise sessions could have enhanced their satisfaction with the program.

### Theme 3: coexisting comorbidities, challenges with mobility and transport got in the way

Despite the positive results experienced by many participants, some of them reported some constraints associated with pre-existing comorbidities that hindered their engagement with the program, including difficulties in performing certain exercises, and continuing the exercise sessions on their own. For example, Tereza had no difficulty in performing the exercises except for one that was prescribed in supine:

*“I only thought it was bad when I had to lie in bed, I never liked doing it. When I lie down I get shortness of breath, I have pulmonary emphysema. I’m asphyxiated, [do you] understand? But for the most part I thought it was wonderful, I had no difficulty doing it.” Tereza*

Antônia also experienced some difficulties in exercises and discussed a lack of confidence to walk by herself during the program due to her knee:

*“They’d give me the explanations, right, and I’d get here and do it. I’d walk for ten minutes in the house, right? And walking, I could never walk because I had a problem with my knee. And I was afraid to walk on my own. And to go for a walk on my own, I’m very nervous, you know, just to leave the house.” Antônia*

Likewise, some participants reported that health problems not only limited their ability to engage in exercises but also made them concerned about developing other health problems due to their age, feeling that comorbidities hindered their long-term progress*.* Francisca experienced intense back pain before starting the program but experienced improvements over time. However, symptoms potentially related to hip arthritis made it difficult for her to continue the exercises, potentially hindering the benefits of the program after its completion:

*“No, I never did it [the exercises] again, because of this pain in my hip. The cartilage, I call it cartilage, I don’t know if it is, I think that was the doctor’s explanation”.* Francisca

Some participants mentioned how other health conditions prevented them from attending a few sessions. Sônia, for instance, said she was unable to attend two out of eight sessions due to being unwell:

*“I think my participation was reasonable, right? I didn’t make many friends, also because it was a short time. We expected it to be longer, and I wasn’t there for the last two, right? Due to health problems. And... but I always enjoyed them, they were very good.” Sônia*

Some participants said they encountered physical barriers, including the presence of stairs within the facilities where the intervention was delivered, which limited their ability to circulate in the environment. Other identified issues related to access to transport, which prompted them to walk to the center where PAT-Back was delivered. Within this context, the sessions’ time and the associated weather conditions–particularly sun exposure and high temperatures in late mornings or afternoons—were considered important barriers:

*“I didn’t like the time of the sessions because the time was cruel for us to come at midday under the hot sun, holy Mary, […] if you have to walk [to the session], then you don’t have that resistance anymore, neither does your skin.”* Helena

Still within the context of factors that impacted participation in exercise, Maria José shared that although she intended to remain active through walking, she did not feel safe to walk in her neighborhood where she lived due to crime:

*“There is a sidewalk, but I’m afraid to go out. Sometimes there are people who walk early in the morning, but I’m afraid to go out.”* Maria José

Taken together, these excerpts highlight that having comorbidities alongside CLBP and facing certain environmental challenges such as lack of safety influenced participants engagement with the program, as well as the sustainability of the changes the program prompted (e.g., engaging in walking to remain active), which could in turn influence the perceived clinical benefits following participation in PAT-Back.

### Theme 4: technology can be both helpful and challenging

There were mixed responses as to the perceived utility of using text messages to prompt them to do the home-based exercise program. While motivational text messages were perceived as beneficial by some, many others highlighted how challenges related to access and digital literacy reduced their potential to motivate adherence to exercise. Yet, some participants thought the text messages were useful for feedback, monitoring, and tailoring the exercises according to their needs:

*“Some days she would send a message ‘‘If you can’t do this exercise, do it like this’’, right? Do less, try a little more’’, you know? [For] everything in life [you] must get feedback.” Célia*

Many participants also felt the messages prompted them to perform the exercises, acting as a reminder of regularity during their daily routine. For example, Claudia discussed how she would become aware of her responsibility when receiving the text messages:

*“Every day the physiotherapist would send a message encouraging us to do the exercise, so it was very good because even if I didn’t want to, because I didn’t used to do it, even if I forgot, the physiotherapist would remind me, you know? It was really good.” Claudia*

While most perceived the text messages as useful, some participants experienced difficulties using texting features on mobile phones, as they were used to using phones solely for phone calls:

*“The only thing I know how to do on my mobile phone is receive and call. I don’t know how to do things like that, let’s say, online, I don’t know about that. Just to receive a message on WhatsApp, make a call, I can’t even write on my mobile. They gave it to me as a present, just to keep me entertained.”* Antônia

*“I’m not, let’s put it this way, working with this [mobile phone], this is a semi-computer. But I haven’t got the hang of it yet, it’s just for me to answer calls or call.” João*

For some, family support was key to overcoming technological barriers and worries about getting in contact with the physiotherapists throughout the week, outside of the group-based sessions. Others expressed frustration over the lack of support from family members when they required help to learn how to use their mobile, exemplifying intergenerational challenges within family and peers:

*“I have a son. He doesn’t like to help me, once he called me stupid because I asked him to help me and he said 'Holy Mary, I already explained it twice!’”. Tereza*

Overall, the excerpts above illustrate a tension– participants were generally positive towards the use of technology, but their perceptions around using mobile phones for texting and low digital literacy hindered its use.

## Discussion

This study investigated older adults’ experiences with an exercise plus education program for CLBP—PAT-Back. Our results underscore how support from peers, family members and physiotherapists fostered participation in the education plus exercise intervention and exercise more broadly. Notably, participants felt compelled to join PAT-Back due to their pain intensity and the difficulties accessing healthcare. At times, environmental factors, comorbidities, issues related to mobility and transport hindered engagement with PAT-Back. Yet, the exercises generally exceeded their expectations and when combined with education seemed to shift their thinking regarding the usefulness of exercise when managing CLBP– from skepticism to perceiving benefits from exercise. The use of text messages was reported as beneficial but challenging for some, often due to low digital literacy or perceptions around the use of mobile phones in general (i.e., the idea mobile phones are used for making calls only). Importantly, participants from PAT-Back are from an underserved area of Northeastern Brazil. To the best of our knowledge, these results add to the literature of CLBP among older adults by providing a nuanced understanding of contextual factors impacting CLBP management among older adults in an underserved community setting.

The first theme highlighted that older adults’ participation in exercise and education programs is intertwined with social support and the attitudes of professionals, peers, family and health systems. The importance given to this multi-layered support system aligns with previous findings in different contexts. Schoeb [26] identified that attributes of daily physical, emotional and financial support from family and friends benefited older adults with CLBP living in Switzerland or Hong Kong. Drawing from their results, the authors of that study argued that cultural contexts should be considered when planning care for this population. We concur and, based on our findings, we suggest that a dynamic interplay between these support systems plays a crucial role in helping older adults to sustain engagement in exercise programs.

The second theme highlighted how participating in the education plus exercise program enabled participants to overcome negative beliefs, changing their perspectives about the importance and usefulness of exercise as a self-management strategy. Similarly, older adults who participated in a program encompassing chiropractic care and home-based exercises expressed that they gained self-efficacy for controlling their spinal pain [18]. Through experimentation, participants from our study contrasted previous fears and beliefs about pain with more positive perspectives and reflected on negative perceptions of pain management influenced by others or personal past experiences. These experiences of seeking treatment and not improving, as well as the experiences of receiving unhelpful advice from healthcare professionals are largely similar to those reported by older Brazilian adults with CLBP who participated in another qualitative study [27]. Yet, despite previous negative experiences, the participants of our study generally perceived the education plus exercise program as worthwhile.

The third theme highlighted participants’ experiences with comorbidities and environmental factors (e.g., weather, unsafe neighborhood) during the program. Although much of the literature does not take these factors into account when implementing exercise interventions for CLBP, our participants’ narratives demonstrated that factors such comorbidities tend to overlap with CLBP and when combined, they hinder exercise participation prior to the program and after the program ceases. This finding adds to the literature on barriers older adults face when attempting to exercise, such as fear of falling, fear of injury and lack of social support [28]. When considered together, the barriers reported here and in previous literature help us to understand why engagement in exercise is so difficult for many older adults with CLBP. For instance, Lansburry [29] explored older adults’ strategies to cope with pain, and analysis of interview data identified that exercise was the least preferred option for care. For some participants, being afraid of falling or having other health issues prevented them from exercising [29]. Taken together, our findings and those of others highlight the importance of addressing barriers to exercise rather than simply telling older adults to exercise to manage CLBP. Within this context, age-related vulnerabilities that can prevent ongoing exercise participation require attention, and exercise interventions targeting individuals from underserved backgrounds also need consideration [30].

Despite the valuable insights facilitated by this investigation, we recognize these results have several limitations. First, these results are based on the very specific context of a unique selection of patients from a feasibility RCT, and this limits the transferability of our findings to the broader population of adults with CLBP. Yet many of our results are likely to be transferrable to countries with healthcare systems like the Brazilian National Health System, where accessibility to care for CLBP is very low. We also interviewed participants after the completion of the feasibility study. Therefore, their perceptions about the program may have been influenced by their ability to remember what the program was like at the time they participated in it. The interviews were conducted 3–6 months after the program ended, which means that participants may have had difficulties to recall certain aspects of the program. While this could have been minimized if they were interviewed sooner, we believe that interviewing participants months after PAT-Back ended enabled us to gain insights into the sustainability of the changes they experience (e.g., about the role of exercise in CLBP, symptom improvement)*.* It is also important to note that most participants were women, which reflects some observations that women are more likely to seek care for pain [31, 32]. While the overrepresentation of women in our sample may be considered a potential lack of diversity in terms of gender, it also provided insights into the feminization of aging in Brazil [33]. It is also important to acknowledge that this study was conducted in Brazil and may not be transferable to the context of older adults who belong to high socioeconomic groups and/or identify with other cultures. Lastly, since two out of the five researchers were involved in the feasibility study [16], it is possible that their involvement in that study may have led to an overly positive analysis of participants’ responses. Therefore, it was important for us to be reflexive about our positionality. To this end, we attempted to provide a balanced analysis of participants’ responses, and discussed their views about aspects of the intervention that could have been better and considered as part as a full large scale RCT (e.g., psychological support, consideration of the time of the day the intervention was delivered).

This study also has strengths. To ensure the credibility of data collection, we conducted pilot testing of interview scripts, followed by team discussions and feedback before conducting the interviews. Our team also engaged in regular discussions and debriefing sessions to enhance the range of interpretations and the depth of our analysis. Finally, although researchers have a background in Physiotherapy, there are diverse research focuses, encompassing expertise in low back pain, physical activity, primary care, qualitative research, older adults, and equity. This provides a comprehensive exploration of data from distinct viewpoints.

Future investigations on conservative interventions for CLBP should incorporate the perceptions and experiences of older adults for both designing and implementing education plus exercise programs. Our results highlighted the unique challenges they face when joining a program, emphasizing the need to frame interventions within their specific context—e.g., considering comorbidities; access to transport; peer, community, and professional support across the continuum of care. Importantly, our findings highlight the need for ongoing exercise programs in the community. Urgent priority should be given to delivering context-tailored interventions that provide access to evidence-based treatment at primary care for older adults with CLBP [4]. Addressing these gaps is particularly important within the context of low-income settings [34], where older adults face socioeconomic challenges that can limit affordability and accessibility to services, making publicly funded community-based programs crucial for reducing the impact of CLBP.

## Conclusion

Participants’ perspectives on participating in an exercise plus education program provided insights about the interrelated biological (e.g. comorbidities), social (e.g. peer and family support) and environmental (e.g., transport, neighborhood safety) factors that may influence older adults with CLBP. Education plus exercise intervention protocols for this population should be tailored at the conception phase to meet their needs, especially those related to social and environmental support to facilitate participation, and sustainable engagement in these programs.

## Data Availability

No datasets were generated or analysed during the current study.

## Appendix 1: Semi-structured interview guide

*Questions about patients’ expectations and perceptions before joining the program:*

1. What did you expect from low back pain treatment you received?
  Follow-up question: What made you enrol in the program?

2. What did you think about physical activity for people with low back pain before undergoing treatment?

*Questions about the experience of treatment for low back pain*

3. What were your thoughts about the education sessions (i.e., the part of the session where the physiotherapists provided information about low back pain)?
  Follow-up questions:
  What did you think about what the physiotherapist shared with you?
  What was it like to try to apply the information you received into your daily life?

4. What did you think of exercise sessions supervised by the physiotherapist?
  Follow-up questions:
  How did you feel during the exercise sessions supervised by the physiotherapist? Was there anything you found difficult? If so, what did you find difficult?
  Was there anything about the exercise sessions you found helpful? If so, what was that?

5. What did you think about doing the exercises at home, on your own?
  Follow-up questions:
  How did you feel about doing the exercises at home on your own?
  Was there anything you found difficult? If so, what did you find difficult?
  Was there anything about the home exercises that you found helpful? If so, what was that?

6. What did you think of the WhatsApp messages you received reminding you to do the exercises?
  Follow-up questions:
  Follow-up questions: How did you feel when you received the WhatsApp messages?
  Was there anything you found difficult about receiving the WhatsApp messages? If so, what did you find difficult?
  Were the WhatsApp messages helpful? If so, how did they help?

*Questions about perceptions after the treatment for low back pain*

7. Now that the program has finished, how would you describe your experience of participating in the program for low back pain?
  Follow-up questions:
  What did you like about the program?
  What did you dislike about the program?

8. What changes did you notice after the program, if any?
9. What are your thoughts about physical activity and low back pain after finishing the program?
10. Can you tell us about your experiences doing the exercises at home now that the program has finished? For instance, are you still doing them? Why/why not?
  Follow-up questions:
  What has made it difficult for you to do the exercises at home?
  What has helped you to do the exercises at home?

11. Is there anything else you would like to share with us?
